# Transcriptomic analyses reveal the potential antibacterial mechanism of citral against *Staphylococcus aureus*

**DOI:** 10.3389/fmicb.2023.1171339

**Published:** 2023-05-12

**Authors:** Zedong Liao, Keshan Lin, Weijiang Liao, Ying Xie, Guoqing Yu, Yan Shao, Min Dai, Fenghui Sun

**Affiliations:** ^1^School of Laboratory Medicine, Chengdu Medical College, Chengdu, Sichuan, China; ^2^Sichuan Provincial Engineering Laboratory for Prevention and Control Technology of Veterinary Drug Residue in Animal-origin Food, Chengdu Medical College, Chengdu, Sichuan, China; ^3^The Second People’s Hospital of Pinghu, Pinghu, Zhejiang, China

**Keywords:** citral, *Staphylococcus aureus*, RNA-sequencing, energy metabolism, reactive oxygen species

## Abstract

**Background:**

The emergence of multi-drug resistant *Staphylococcus aureus* (*S. aureus*) has posed a challenging clinical problem for treating its infection. The development of novel or new antibacterial agents becomes one of the useful methods to solve this problem, and has received more attention over the past decade. Citral is reported to have antibacterial activity against *S. aureus*, but its mechanism is yet entirely clear.

**Methods:**

To reveal the antibacterial mechanism of citral against *S. aureus*, comparative transcriptomic analysis was carried out to analyze the gene expression differences between the citral-treated and untreated groups. The changes of protein, adenosine triphosphate (ATP) and reactive oxygen species (ROS) content in *S. aureus* caused by citral were also examined.

**Results:**

Six hundred and fifty-nine differentially expressed genes were obtained according to the comparative transcriptomic analysis, including 287 up-regulated genes and 372 down-regulated genes. The oxidoreductase activity and fatty acid degradation pathway were enriched in up-regulated genes, and ribosome and *S. aureus* infection pathway were enriched in down-regulated genes. Meanwhile, physiological trials revealed a decline in ATP and protein levels, but an increase in ROS content within the citral-treated group. Thus, it can be inferred that the antibacterial effects of citral against *S. aureus* were likely due to its ability to decrease ATP content by down-regulating ATP synthase genes (*atpD* and *atpG*), reduce protein content, induce cell membrane and cell wall damages, accumulate ROS, and down-regulate virulence factor genes to reduce pathogenicity.

**Conclusion:**

These findings revealed the antibacterial mechanism of citral was likely a type of multi-target mode that affected multiple molecular processes in *S. aureus*, which lays the groundwork for further exploitation of citral as a therapeutic candidate against *S. aureus* infections.

## 1. Introduction

*Staphylococcus aureus* (*S. aureus*) is a major human pathogen that causes skin, soft tissue, respiratory, joint, and other infection (Cheung et al., 2021). It is also responsible for foodborne intoxication worldwide (Hennekinne et al., 2012). According to the reports, *S. aureus* had a fatality rate equivalent to HIV/AIDS, tuberculosis, and viral hepatitis in the United States (van Hal et al., 2012). With the emergence of multi-drug resistant strains, treating *S. aureus* infection has become a challenging clinical problem, which is increasingly threatening public health and well-being (Tarai et al., 2013). To tackle the situation, the discovery of new drug candidates has received more attention in the last decade.

Citral (3,7-dimethyl-2,6-octadienal) is a monoterpenic aldehyde formed by a natural mixture of geranial (trans-citral) and neral (cis-citral). It is found in various plants, such as myrtle trees, bergamot, melissa, lemongrass, and verbena (Dudai et al., 2005; Shi et al., 2016). Studies showed that citral had several pharmacological activities such asanti-inflammatory, anticancer, analgesic, antispasmodic, antiparasitic, and immunomodulatory action (Bao et al., 2015; Martins et al., 2017; Oliveira et al., 2021). Besides that, several studies have reported antimicrobial effects of citral against several pathogenic bacteria (Somolinos et al., 2010; Ouyang et al., 2018; Qian et al., 2020). Our previous studies found that citral exhibited obvious antibacterial activity against *S. aureus* in vitro (Fenghui et al., 2018); and obvious curative effect in a murine infection model (Long et al., 2019). Several studies also were conducted to uncover the antibacterial mechanism of citral. Qian et al. (2020) carried out a confocal laser scanning microscopy analysis and found citral might disrupt the cell membrane integrity of carbapenem-resistant *Enterobacter cloacae*. The findings from the reactive oxygen species (ROS) content assay and proteomic analysis revealed that citral induces substantial ROS accumulation and results in the impairment of oxidative phosphorylation in *Penicillium digitatum* (Ouyang et al., 2018). Gupta et al. (2017) found citral could lead to an increase in the extracellular nucleic acid content of *S. aureus* and speculated that citral could damage the cell membrane. Although several studies found citral induced cell membrane and cell wall damages, and metabolic changes, the antibacterial mechanisms of citral have not been fully characterized and need further investigation.

With the advent of next-generation sequencing and the development of various bioinformatics tools, RNA sequencing has played an important role in exploring gene expression involved in pathogenic response to various antimicrobial stress, recently (Sudhagar et al., 2018; Li et al., 2021). Here, we carried out a transcriptomic analysis to characterize alterations of gene expression. Meanwhile, we conducted some physiological trials to evaluate the content changes of protein, adenosine triphosphate (ATP), and ROS caused by citral. The results obtained from this study will provide valuable insights into the antibacterial mechanisms of citral against *S. aureus*, which can serve as a basis for further research on antimicrobial agents and targets.

## 2. Materials and methods

### 2.1. Bacterial strain

*S. aureus* strain NCTC8325 was obtained from the China Center of Industrial Culture Collection and stored at −80°C. The bacteria were plated on the nutrient agar (Aoboxing, Beijing, China) plates at 37°C overnight, and a single colony was picked up for the following study.

### 2.2. The antibacterial activity of citral

The antibacterial activity of citral was characterized by minimum inhibition concentration (MIC) and minimum bactericide concentration (MBC). The MIC was determined by the broth dilution method in 96-well plates based on the guidelines from the Clinical and Laboratory Standards Institute, according to the methods described by Ronco et al. (2020). To determine MBC, 10 μL cultures from each well were inoculated on MHA (Solarbio, Beijing, China) plates and incubated at 37°C for 16–20 h. The MIC was defined as the lowest concentration of citral with no visible bacterial growth. The MBC was defined as identified as the lowest concentration of citral at which no colony growth was observed on MHA plates.

### 2.3. Growth curve

A single colony of *S. aureus* was inoculated into 0.9% sterile saline and adjusted to 0.5 McFarland. Subsequently, the bacterial solution was added into MHB (Solarbio, Beijing, China) medium containing citral (Sigma-Aldrich, St Louis MO, United States) with the final concentration of 1/8 MIC, 1/4 MIC, and 1/2 MIC, respectively. The cells were incubated at 37°C and 200 rpm/min for 24 h. Samples were taken every 2 h, and the optical density at 600 nm (OD_600_) was recorded by Varioskan Flash (Thermo Fisher Scientific, United States). The samples without citral treatment were used as the control group.

### 2.4. Sample preparation for RNA sequencing

To obtain the gene expression changes of *S. aureus* caused by citral, the transcriptomic analysis was conducted according to Hua et al. (2019). In short, *S. aureus* was grown to mid-log phase (OD_600_ = 0.6), then the cells were treated with citral (1/2 MIC) at 37°C for 1 h. The cells without citral treatment were considered as control. The cells were collected by centrifugation and rinsed twice with sterile water. The pellets were immediately frozen in liquid nitrogen and sequenced on the NovaSeq 6,000 by Novogene Co., Ltd. (Beijing, China). All experiments were performed in triplicates.

### 2.5. RNA libraries and sequencing

Total RNA was isolated from cell pellets by RNAprep Pure Cell/Bacteria Kit (Tiangen, Beijing, China). The isolated RNA was then qualified by agarose gel electrophoresis and RNA Nano 6,000 Assay Kit on the Bioanalyzer 2,100 system (Agilent Technologies, CA, United States). The purified mRNA was then acquired by removing the rRNA through probes. The library was constructed and assessed on the Agilent Bioanalyzer 2,100 system (Parkhomchuk et al., 2009), subsequently. Illumina NovaSeq 6,000 was used to sequence the library, and 150 bp paired-end reads were generated. Clean data were obtained by removing reads containing adapter, reads containing N base, and low-quality reads from raw data. Simultaneously, Q_20_ (percentage of bases with a Phred value >20), Q_30_ (percentage of bases with a Phred value >30), and GC content of the clean data were calculated. The Raw sequence data reported in this paper have been deposited in the Genome Sequence Archive in National Genomics Data Center, China National Center for Bioinformation/Beijing Institute of Genomics, Chinese Academy of Sciences (GSA: CRA009337), which are publicly accessible at https://ngdc.cncb.ac.cn/gsa.

### 2.6. RNA-sequencing data analysis

The clean reads were aligned and annotated to reference genome *S. aureus* subsp. aureus (GenBank Accession No. NC_007795.1) using the software of Bowtie2 (Langmead and Salzberg, 2012). Then the reads numbers mapped to each gene were counted by Subread (Liao et al., 2014). The Differential expression analysis was performed using the R package of DESeq (Love et al., 2014). The *value of p*s were adjusted using the Benjamini and Hochberg method. Genes exhibiting an adjusted *value of p* <0.05 and an absolute log2 (fold-change) ≥ 1.5 were classified as differentially expressed genes (DEGs) (Okeeffe et al., 2014). In addition, to determine the enriched biological functions and pathways, Gene Ontology (GO) and Kyoto Encyclopedia of Genes and Genomes (KEGG) enrichment analyses of DEGs were conducted by the R package: clusterProfiler (Yu et al., 2012).

### 2.7. Quantitative real-time PCR (qRT-PCR)

To verify the RNA-sequencing data, the relative expression levels of genes were confirmed by qRT-PCR. RNA samples were obtained in the same way as the preparation of RNA-Sequencing. Total RNA was extracted with RNAprep Pure Cell/Bacteria Kit (Tiangen, Beijing, China). Then, first-strand cDNAs were synthesized using FastKing RT Kit with gDNase (Tiangen, Beijing, China). The procedure was performed with Genious 2X SYBR Green Fast qPCR Mix (No ROX) (ABclonal, Wuhan, China) following the manufacturer’s protocol with the CFX Connect real-time PCR system (BIO-RAD). The sequences of all the primers used are listed in Supplementary Table S1. The gene *tpi* was used as a housekeeping gene (Szalus-Jordanow et al., 2018). The relative gene expression was calculated by the 2^−ΔΔCT^ method.

### 2.8. Determination of protein content

*S. aureus* protein content was determined using the reported method with a few modifications (Wang et al., 2020). Briefly, the mid-log phase cells were exposed to different concentrations of citral (1/4 MIC, 1/2 MIC, and MIC) for 0 and 1 h. *S. aureus* without citral was treated in the same way and used as the controls. The treated samples were collected by centrifugation, and then were washed and resuspended in PBS buffer. The proteins were extracted from the samples and detected by the bicinchoninic acid (BCA) protein Assay Kit (Solarbio, Beijing, China).

### 2.9. Determination of ATP content

The changes in intracellular and extracellular ATP concentration in *S. aureus* (mid-log phase) were detected as described by Lan et al. (2021), with slight modifications. In brief, the bacteria cultures were centrifuged at 10,000 × g for 5 min after administration with citral (1/4 MIC, 1/2 MIC and MIC) for 1 h. The content of intracellular and extracellular ATP was measured by the enhanced ATP Assay Kit (Beyotime, Shanghai, China) according to the manufacturer’s instructions. The luminescence intensity was detected by the Flexstation 3 microplate reader (Molecular Devices, United States). The bacterial cells that were not treated with citral were similarly processed and served as controls.

### 2.10. Determination of ROS content

ROS content was determined according to the methods of Kang et al. (2019). In short, mid-log phase cultures of *S. aureus* cells were treated with citral at final concentrations of 1/4 MIC, 1/2 MIC, and MIC at 37°C for 1 h. Then the collected cells were washed, resuspended, and adjusted to 0.5 McFarland in PBS buffer. The fluorescence probe DCFH-DA in the ROS Assay Kit (Beyotime, Shanghai, China) was used to characterize ROS content according to the manufacturer’s instructions. The fluorescence intensity was measured on the NovoCyte^™^ flow cytometer (ACEA, United States) with the excitation/emission setting at 488/525 nm. Cells without the citral treatment were used as controls.

### 2.11. ROS quenching experiments

The thiourea, a specific ROS scavenger, was added to the medium to further confirm the oxidative damage of citral to *S. aureus*. In brief, the bacterial with citral (1/2 MIC) in the presence or absence of thiourea (Macklin, Shanghai, China) was incubated at 37°C and 200 rpm/min for 6 h. The OD_600_ value of samples was recorded by Varioskan Flash (Thermo Fisher Scientific, United States).

### 2.12. Statistical analysis

All experiments in this study were performed in triplicates. The study data were expressed as the mean and standard deviation (mean ± standard deviation). Statistical analysis was conducted using a one-way analysis of variance with GraphPad Prism 8.0 software. In all instances, a *p*-value less than 0.05 was deemed statistically significant. The statistically significant differences were indicated by asterisks (*p ≤ 0.05; **p ≤ 0.01; ***p ≤ 0.001).

## 3. Results

### 3.1. Antibacterial activity of the citral against *S. aureus*

**Figure 1:**
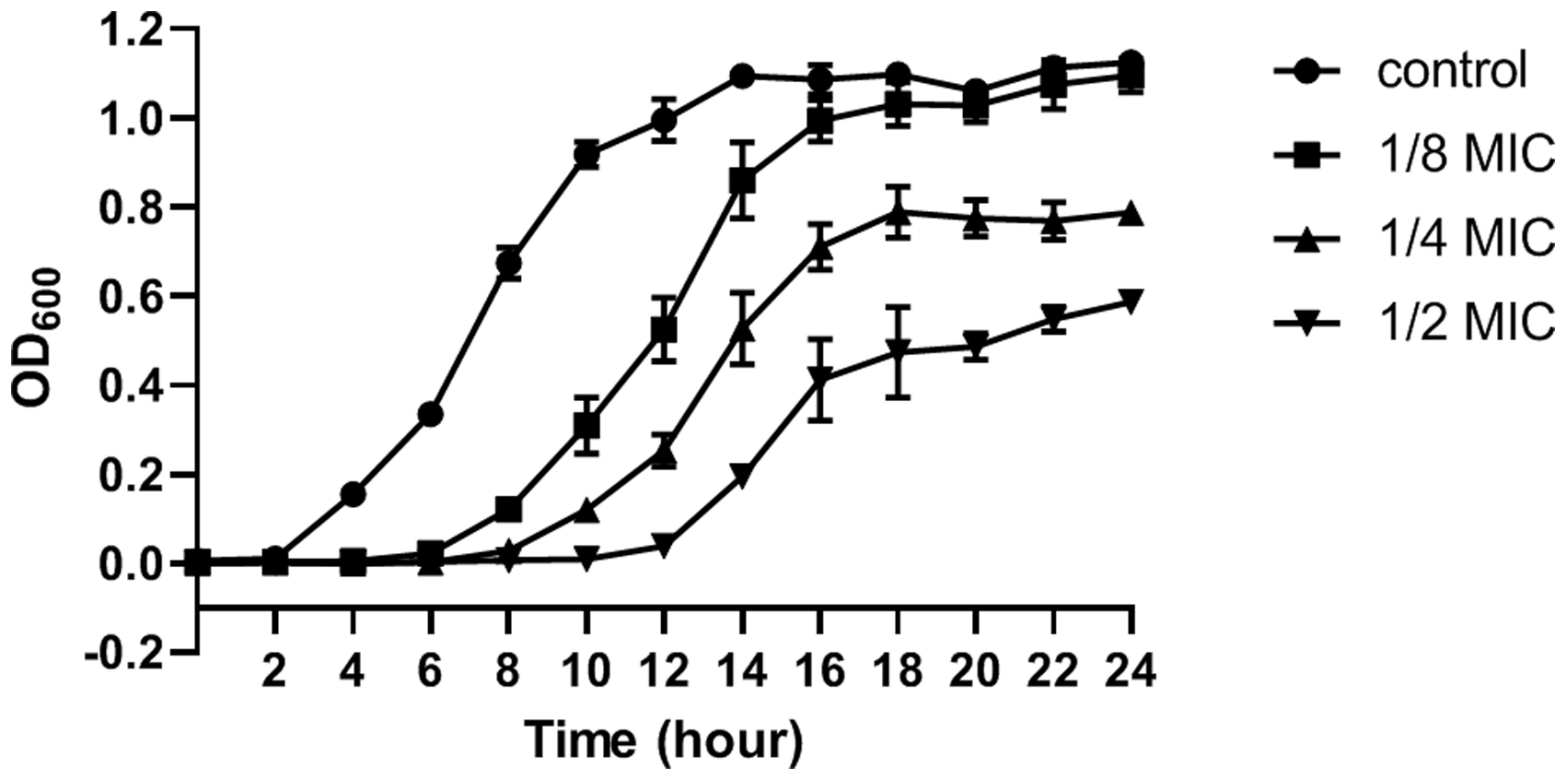
The growth curve (optical density at 600 nm) of *S. aureus* after treatment with citral: control (closed circles), 1/8 MIC (closed squares), 1/4 MIC (closed triangles), 1/2 MIC (inverted closed triangles). The presented results are the mean of three independent experiments, with error bars denoting standard deviation.

The MIC and MBC of citral against *S. aureus* was 695 μg/mL and 1.39 mg/mL, respectively. The effect of citral on the growth of *S. aureus* was shown in Figure 1. The untreated *S. aureus* managed to reach the log phase after 2 h incubation at 37°C, reach the stationary phase and biggest biomass (OD_600_ = 1.12 ± 0.01) after 14 h incubation. By contrast, the growth of *S. aureus* was inhibited after citral treatment and the lag phase of *S. aureus* was prolonged with the increasing concentration of citral. The biggest biomass of the citral-treated samples was lower than those of the control samples. These results indicated that citral could inhibit the growth of *S. aureus*, and the inhibitory effect rised with the increasing of citral concentration within the test range. Citral (1/2 MIC) showed the strongest inhibitory effect on *S. aureus* and was chosen as the working concentration for subsequent transcriptomic investigation.

### 3.2. RNA-sequencing analysis

#### 3.2.1. RNA-sequencing and quality control

RNA sequencing generated 21,008,116 and 24,917,534 raw reads in the citral-treated and control samples, respectively. A total of 45,663,028 clean reads were produced after removing low-quality reads. The Q_30_ percentage of each library was more than 90%. All clean reads were mapped to the reference genome of *S. aureus*, and the total mapping ratio ranged from 99.16 to 99.43% (Supplementary Table S2). Meanwhile, the mRNA expression exhibited high repeatability with correlation coefficients more than 0.934 (Supplementary Figure S1) within the biological triplicates of each group. All these indicated that high-quality sequencing data were obtained and suitable for further analysis.

#### 3.2.2. Transcriptional profile analysis

**Figure 2:**
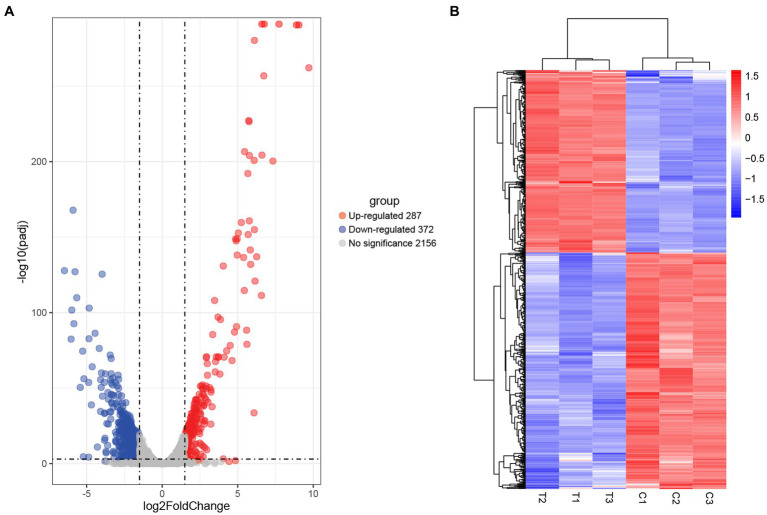
Screening of DEGs in *S. aureus* induced by citral treatment: **(A)** volcano plots and **(B)** hierarchical cluster analysis (heatmaps) of the DEGs between citral-treated samples (T1–T3) and controls (C1–C3). Statistically significant DEGs were defined with an adjusted *p* (*p*-adj) value < 0.05 and absolute log2FoldChange ≥ 1.5 as the cutoff threshold.

Comparative transcriptomic analysis was performed to understand the gene expression variations between citral-treated and untreated groups. As compared to the control group, 659 DEGs were identified. Of these, 287 were up-regulated, and 372 were down-regulated (Figure 2). The top ten up-regulated genes (Table 1) mainly related to stress response-related genes in *S. aureus*, for example, *vraX* (encoding C1q-binding complement inhibitor), *cwrA* (encoding cell wall inhibition responsive protein), *dnaK* (encoding molecular chaperone), *grpE* (encoding heat shock protein), *mcsB* (encoding guanido phosphotransferase), and *clpC* (encoding endopeptidase) (Wozniak et al., 2012; Cuaron et al., 2013). Meanwhile, genes that have important physiological roles, such as *argG* (encoding argininosuccinate synthase), *pflA* (encoding pyruvate formate-lyase 1 activating enzyme) and *pflB* (encoding formate acetyltransferase), were down-regulated (Table 1).

**Table 1:** The top ten up- and down- regulated DEGs.

Category	Gene_id	log2 fold change	*p*-*adj*	Gene_name	Gene_description
Up-regulated	SAOUHSC_02893	9.707596956	6.929E-263	–	DUF896 domain-containing protein
	SAOUHSC_00561	9.040725294	0	vraX	C1q-binding complement inhibitor
	SAOUHSC_02892	8.903467143	0	–	hypothetical protein
	SAOUHSC_02866	7.489908404	0	–	fatty acid efflux MMPL transporter
	SAOUHSC_02872	7.334065647	4.3044E-201	cwrA	cell wall inhibition responsive protein
	SAOUHSC_01683	6.724355817	1.4228E-257	dnaK	molecular chaperone
	SAOUHSC_01684	6.604499429	4.8952E-205	grpE	heat shock protein
	SAOUHSC_02824	6.572427919	4.4638E-112	–	alpha/beta hydrolase
	SAOUHSC_00504	6.528347636	0	mcsB	ATP:guanido phosphotransferase
	SAOUHSC_00505	6.484127314	0	clpC	endopeptidase
Down-regulated	SAOUHSC_02671	−6.471783081	1.611E-128	narT	nitrate transporter
	SAOUHSC_00899	−6.02947604	3.46374E-83	argG	argininosuccinate synthase
	SAOUHSC_00188	−5.983619288	2.0165E-102	pflA	pyruvate formate-lyase 1 activating enzyme
	SAOUHSC_00187	−5.900147115	1.4499E-168	pflB	formate acetyltransferase
	SAOUHSC_02685	−5.845924926	2.22966E-93	–	sirohydrochlorin ferrochelatase
	SAOUHSC_02681	−5.770892267	8.0905E-128	–	nitrate reductase subunit alpha
	SAOUHSC_02680	−5.655444057	1.3733E-110	–	nitrate reductase subunit beta
	SAOUHSC_00131	−5.423736733	3.31075E-51	–	YbaN family protein
	SAOUHSC_02679	−5.263068749	3.38926E-75	–	respiratory nitrate reductase subunit delta
	SAOUHSC_02645	−5.222935859	2.92178E-05	–	LytTR family DNA-binding domain-containing protein

#### 3.2.3. Go functional enrichment analysis

**Figure 3:**
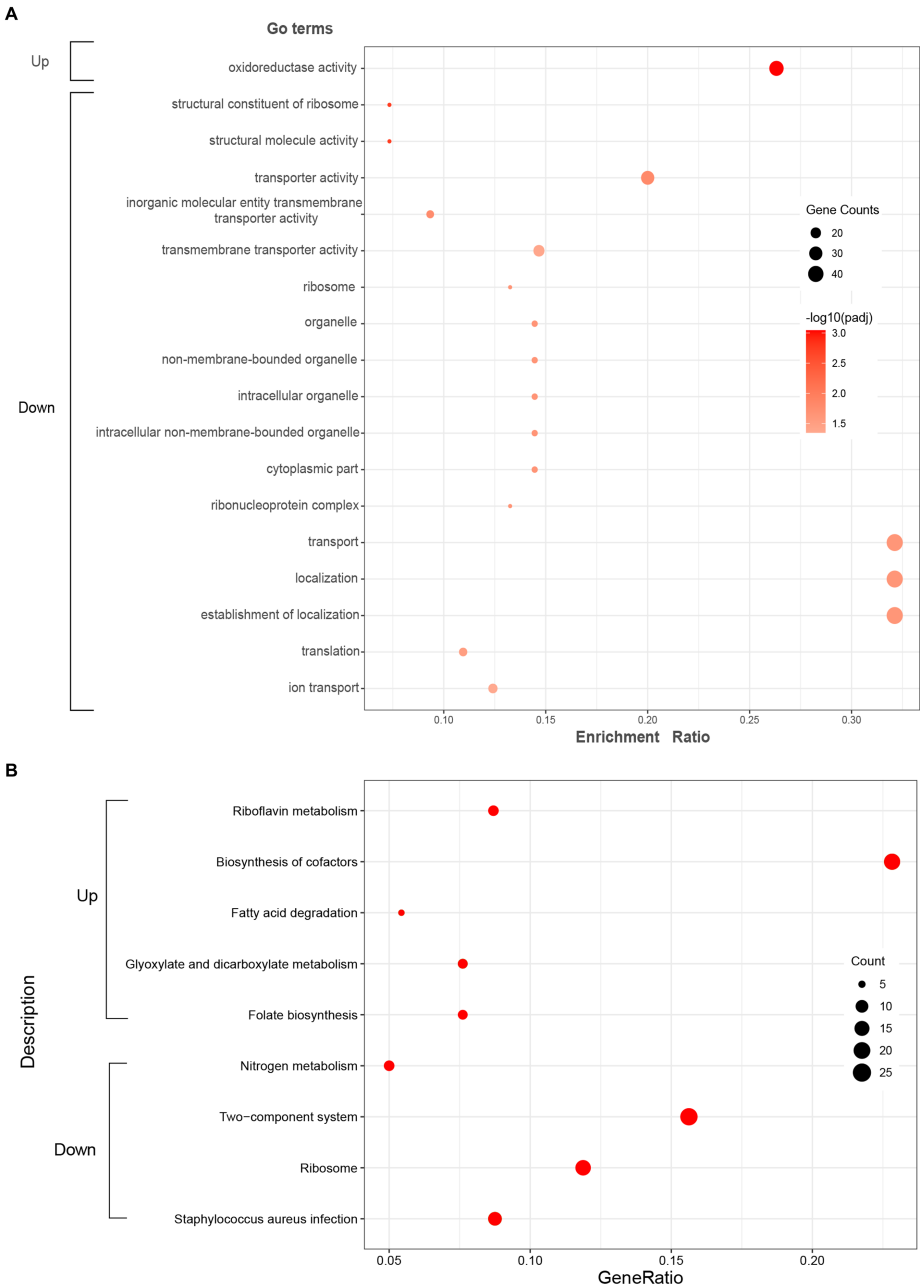
Significantly enriched GO terms **(A)** and KEGG pathways **(B)** of the up-regulated and down-regulated DEGs. Red color in KEGG pathways **(B)** represents *p*-adj < 0.05.

GO enrichment analysis was further performed to understand the biological significances of the DEGs. The significantly enriched (*p*-adj < 0.05) GO terms of the up-regulated and down-regulated DEGs were shown in Figure 3A. For up-regulated DEGs, the significantly enriched GO term was oxidoreductase activity (GO: 0016491) which is classified to molecular functions ontology. Further, 5 terms for biological processes, 7 terms related to cellular components, and 5 terms for molecular functions were significantly enriched in the down-regulated DEGs. Among these terms, the translation (GO: 0006412) and ribosome (GO: 0005840), which are related to protein biosynthesis, were significantly enriched.

#### 3.2.4. KEGG pathway enrichment analysis

KEGG enrichment analysis was executed to analyze the metabolic pathways associated with up-regulated and down-regulated DEGs. The enriched (*p*-adj < 0.05) KEGG pathways were shown in Figure 3B. In up-regulated DEGs, KEGG pathways corresponding to riboflavin metabolism (sao00740), biosynthesis of cofactors (sao01240), fatty acid degradation (sao00071), glyoxylate and dicarboxylate metabolism (sao00630), and folate biosynthesis (sao00790) were significantly enriched, which were mostly related to energy metabolism. In addition, KEGG pathways corresponding to nitrogen metabolism (sao00910), two-component system (sao02020), ribosome (sao03010), and *Staphylococcus aureus* infection (sao05150) were significantly enriched in down-regulated DEGs.

#### 3.2.5. Transcriptional alterations of protein biosynthesis induced by citral

As displayed in Supplementary Table S3, the ribosome pathway was significantly enriched in the down-regulated DEGs. Nineteen out of fifty-five genes encoding ribosomal structural proteins, comprising six 30S proteins and 13 50S proteins, were down-regulated following citral treatment. Further analysis found that the genes (*thrS*, *serS*, *trpS* and *leuS*) which encode threonyl-tRNA synthase, seryl-tRNA synthetase, tryptophanyl-tRNA synthetase and leucyl-tRNA synthetase were also down-regulated by citral. Therefore, transcriptomic analysis indicated that citral might inhibit the protein biosynthesis by downregulating the expression of the ribosomal structural protein and aminoacyl-tRNA synthase genes.

**Figure 4:**
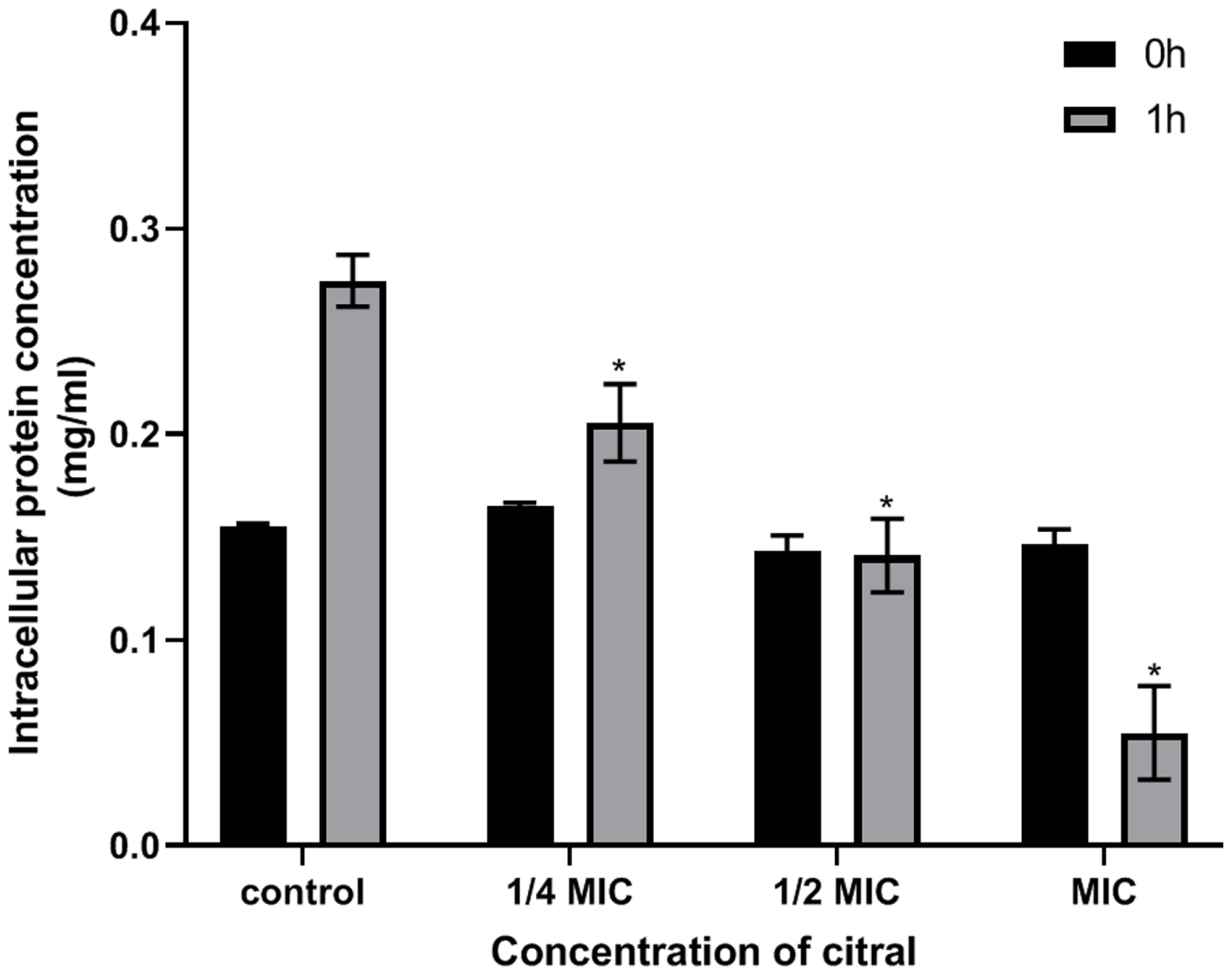
Changes of protein content in *S. aureus* following treatment with citral.

The results of protein content determination also found that citral could decrease the cellular protein content of *S. aureus*. As shown in Figure 4, the cellular protein content of *S. aureus* decreased from 0.27 ± 0.01 to 0.21 ± 0.02, 0.14 ± 0.02 and 0.09 ± 0.00 mg/mL, respectively, when the cell was treated with citral (1/4 MIC, 1/2 MIC, and 1 MIC) for 1 h.

#### 3.2.6. Transcriptional signature of inhibition of ATP biosynthesis by citral

The fatty acid degradation pathway was significantly enriched (*p*-adj < 0.05), in which five genes including *aldA* gene (encoding aldehyde dehydrogenase) were up-regulated (Supplementary Table S3). Besides that, the gene (*SAOUHSC_00707*) encoding phosphofructokinase-1, an important rate-limiting enzyme in the glycolytic pathway (Hon et al., 2022), was significantly up-regulated (3.57-fold). The genes (*SAOUHSC_01802*, *icd* and *SAOUHSC_01614*) encoding the rate-limiting enzymes in the tricarboxylic acid (TCA) cycle were up-regulated. The gene *sucD* encoding the succinyl-CoA synthetase subunit alpha which involved only substrate-level phosphorylation in the TCA cycle (Huang and Fraser, 2020) was up-regulated (3.00-fold). The glycolysis, TCA cycle, and oxidative phosphorylation are the three main ATP-producing pathways in the cell (Fernie et al., 2004). The enriched fatty acid pathway and up-regulated genes encoding for key enzymes in the three main ATP-producing pathways indicated that more ATP were needed to support the growth of *S. aureus*.

**Figure 5:**
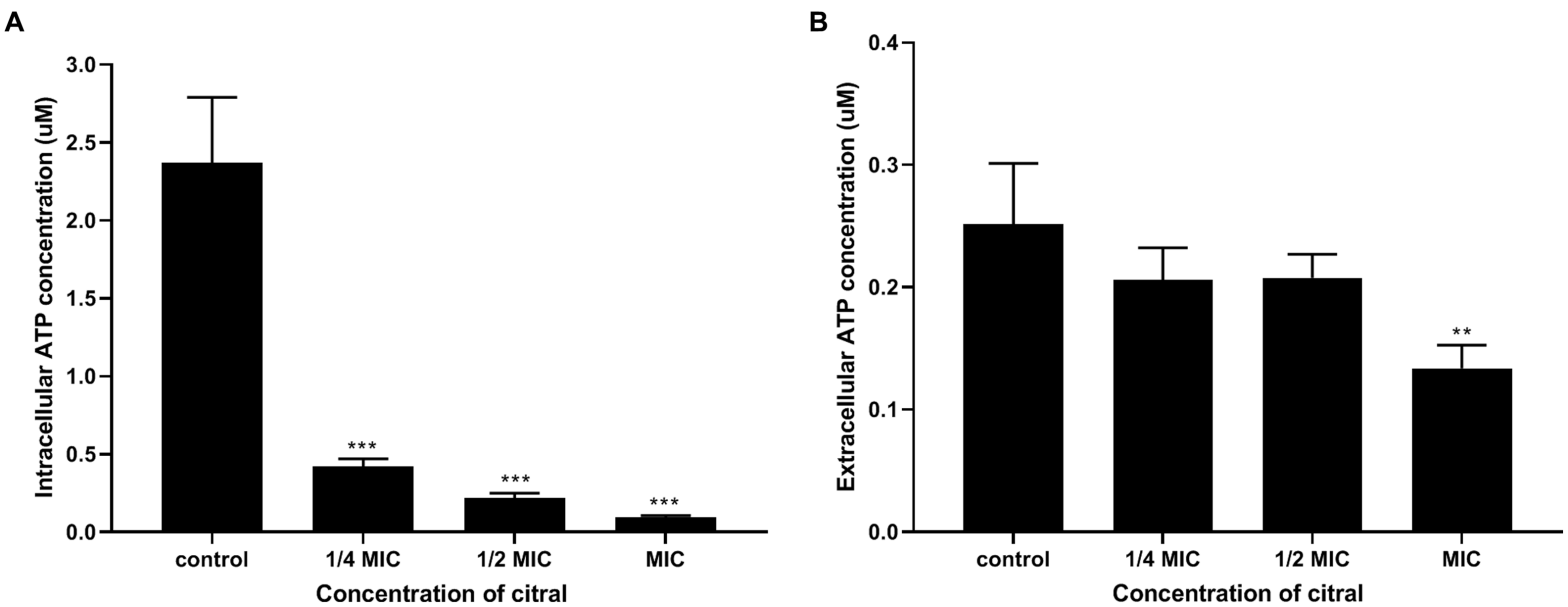
Intracellular **(A)** and extracellular **(B)** ATP contents of *S. aureus* after being treated with citral for 1 h. Values represent the means of three independent experiments.

The results of ATP content determination found that the intracellular and extracellular ATP content decreased after citral treatment. As shown in Figure 5, the level of intracellular ATP of *S. aureus* decreased significantly (*p* ≤ 0.001). The original ATP contents of *S. aureus* was 2.37 ± 0.42 μmol/L. After exposure to citral at different concentration (1/4 MIC, 1/2 MIC, and MIC), the ATP contents of *S. aureus* reduced to 0.42 ± 0.05, 0.22 ± 0.03 and 0.09 ± 0.01 μmol/L, respectively. When it terms to the extracellular ATP content, there was no significant difference in extracellular ATP contents among the control group, 1/4 MIC and 1/2 MIC group, but a 1.92-fold reduction in the MIC group compared with the control group. In conclusion, citral can reduce intracellular ATP content in *S. aureus*, which is unlikely to be caused by leakage to the outside of the cell.

Most of the ATP in the cell is produced by ATP synthase (Nirody et al., 2020). DEGs analysis found that two genes (*atpD* and *atpG*) encoding F0F1 ATP synthase subunits were down-regulated 3.22- and 3.34-fold, respectively (Supplementary Table S3), which implied that citral might inhibit ATP biosynthesis.

#### 3.2.7. Transcriptional signature of ROS elevation and oxidative damage induced by citral

The production of ROS is a natural side effect of aerobic respiration (Imlay and Fridovich, 1991). As described above, the increase of acetyl-CoA and over-expressed genes encoding the rate-limiting enzymes in the TCA cycle suggested that citral might induce the generation of excessive ROS.

**Figure 6:**
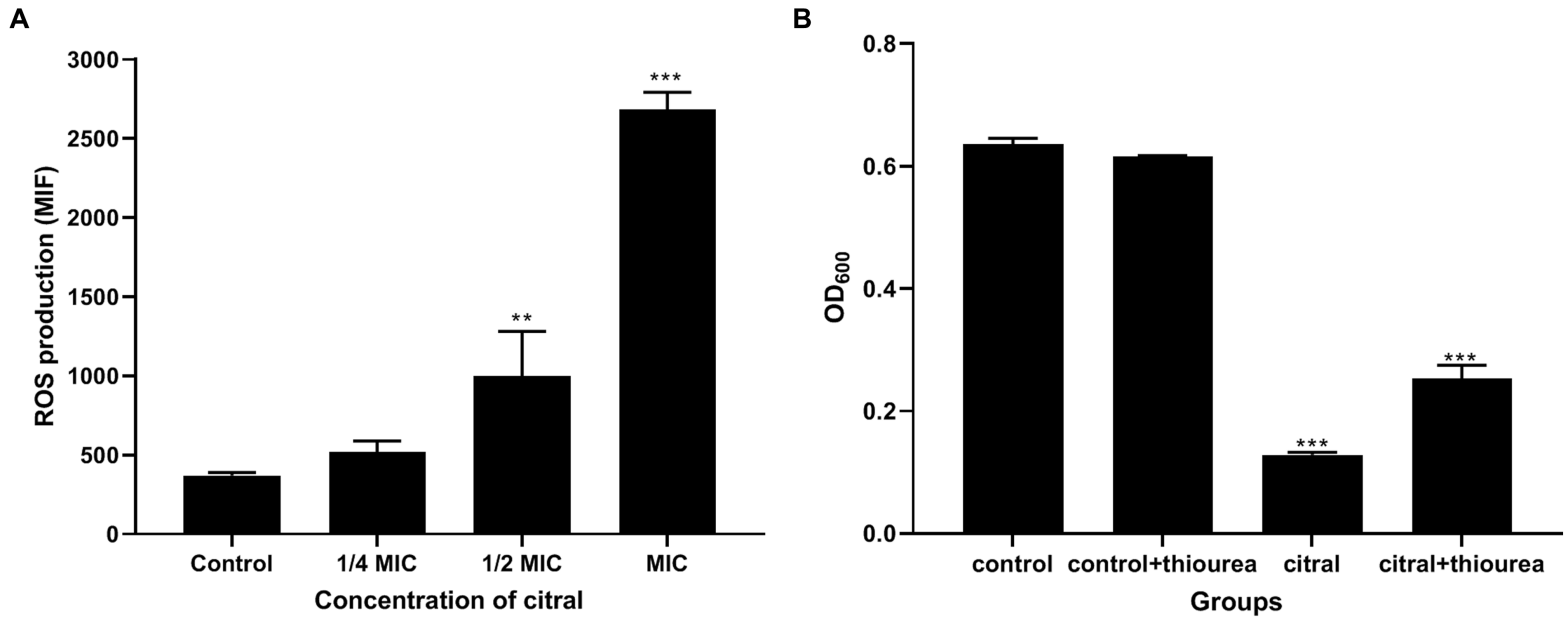
Effect of citral on ROS of *S. aureus*. **(A)** Intracellular ROS production of *S. aureus* cells treated with citral for 1 h shown as mean fluorescence intensity (MFI) values. **(B)** ROS quenching experiments of *S. aureus* treated with citral (1/2 MIC) in the presence or absence of 20 mM/L thiourea. Data are means ± standard deviations.

The above supposition was confirmed by the determination of the ROS content. As shown in Figure 6, the fluorescence intensity of *S. aureus* treated with citral (1/2 MIC and MIC) was much (*p* ≤ 0.01) higher than that of the control, which means the ROS increased in *S. aureus* after citral treatment. The subsequent ROS quenching experiment also confirmed the ROS damage caused by citral. Thiourea, a ROS scavenger was added to the medium, and the results showed that the OD_600_ values in the citral-thiourea treated groups were much higher than those in the citral-only groups, indicating that bacterial growth was partially recovered in the citral-thiourea treatment group.

Further comparative transcriptomic analysis found that several antioxidant genes, such as *sodA* (encoding superoxide dismutase), *katA* (encoding catalase), and *ahpC* (encoding alkyl hydroperoxide reductase), were also up-regulated (Supplementary Table S3), which might be the response of bacteria to the elevation of ROS.

Oxidative stress caused by ROS can result in damage to both the backbone and bases of nucleic acids (Fasnacht and Polacek, 2021). In this study, several genes related to DNA repair were also found to be differentially expressed (Supplementary Table S3.). The genes of *SAOUHSC_00507*, *SAOUHSC_01469*, and *uvrC*, which encodes DNA repair protein, endonuclease III, and excinuclease ABC subunit C, respectively (Riordan et al., 2011; Wang, Y. et al., 2021), were up-regulated. Simultaneously, genes encoding recombination protein (*recR*), primosomal protein N (*SAOUHSC_01179*), and ATP-dependent DNA helicase (*recG*) in homologous recombination pathway were up-regulated. In addition, the genes *ung* and *rnhC*, encoding uracil-DNA glycosylase and ribonuclease H III were down-regulated.

#### 3.2.8. DEGs associated with the cell membrane and cell wall

As shown in Supplementary Table S3, the genes *vraX*, *vraS*, and *vraR* responding to the cell wall stress stimulon (Mccallum et al., 2011; Cuaron et al., 2013) were significant up-regulated by 526.66-, 13.08- and 13.22-fold, respectively, indicating citral might induce cell wall damage to *S. aureus*. Furthermore, the analysis of related DEGs found that the genes (*plsY* and *SAOUHSC_01837*) in the biosynthesis pathway of phosphatidic acid, an essential precursor of membrane phospholipids (Yao and Rock, 2013), were down-regulated by 6.25- and 8.49-fold, respectively. In addition, the genes *scdA* and *tarA*, encoding cell wall biosynthetic protein and teichoic acid biosynthesis protein, were down-regulated 28.45-fold and 3.11-fold, respectively.

#### 3.2.9. DEGs associated with virulence factor

The *Staphylococcus aureus* infection pathway was significantly enriched (*p*-adj < 0.05) in the down-regulated DEGs after citral treatment, which included a large number of genes related to bacterial virulence factor (Supplementary Table S3). The cytolytic toxins genes of *hly* (encoding alpha-hemolysin) and *hld* (encoding delta-hemolysin) were down-regulated 9.49-fold and 3.31-fold, respectively. The surface proteins genes of *spa* (encoding *staphylococcus aureus* protein A) and *sbi* (encoding immunoglobulin G-binding protein) were down-regulated 5.92-fold and 5.90-fold, respectively. Genes encoding enzymes associated with virulence factors, such as *nuc* (encoding thermonuclease) and *SAOUHSC_02171* (staphylokinase), were down-regulated 2.83- and 5.36-fold, respectively. In addition, virulence genes (Kong et al., 2016; Hua et al., 2019; Singh and Phukan, 2019) of *clfB* (encoding clumping factor B), *sdrC*, *sdrD* and *fib* (encoding fibrinogen-binding protein), *cspA* (encoding cold shock protein), *sspA* (encoding glutamyl endopeptidase) and *sspB* (encoding cysteine protease) were also down-regulated.

### 3.3. Validation of DEGs by qRT-PCR

Nine DEGs (*ribBA*, *icd*, *dnaK*, *clpP*, *argG*, *qoxA*, *hly*, *scdA*, and *lrgA*) were randomly selected for qRT-PCR analysis to validate the reliability of the RNA-sequencing results. Similar trends were found between qRT-PCR and transcriptomic data (Supplementary Figure S2; Supplementary Table S4).

## 4. Discussion

Citral exhibits antibacterial activity against many bacteria, including *Cronobacter sakazakii*, *Escherichia coli* and carbapenem-resistant *Enterobacter cloacae* (Somolinos et al., 2010; Shi et al., 2016; Sun et al., 2019; Prakash and Vadivel, 2020; Qian et al., 2020; Selvaraj et al., 2020). Previous studies have found that the citral exhibited in vitro and in vivo anti-*S. aureus* activities (Gupta et al., 2017; Long et al., 2019). Yet, the underlying mechanisms of citral against *S. aureus* remain unclear. The present work compared the gene expression profile and biochemical changes between citral-treated and control groups to elucidate the antibacterial mechanism of citral. Based on our analysis, we found that the DEGs were mainly involved in ATP-related energy metabolism, protein biosynthesis, ROS accumulation, cell membrane, cell wall, and *S. aureus* infection.

ATP is critical for bacterial growth and cell division (Wang, Y. et al., 2021). In this study, we found the cellular ATP content was decreased after citral administration. It is well known that the F1 region in F1F0 ATP synthase is composed of five subunits (*α*, *β*, *δ*, *γ*, and *ε*), and ATP is synthesized from the *α*3*β*3-hexamer by rotating the *γ*-*ε* pair (Santana et al., 1994). Transcriptional analysis showed that the encoding genes of subunit *β* (*atpD*) and *γ* (*atpG*) were suppressed, indicating that citral might reduced ATP content by inhibiting ATP biosynthesis.

When the cellular ATP content declines, bacteria typically modulate their metabolic pathways through two ways: either by inhibiting energy-consuming pathways to conserve energy or by enhancing energy-producing pathways to generate more ATP. It is well-known that protein biosynthesis uses most of the cellular ATP (Pontes et al., 2015). In this paper, numerous genes encoding ribosomal protein (e.g., *rplN*, *rpsS*) and aminoacyl-tRNAs synthetase (e.g., *thrS*, *serS*) were down-regulated after citral treatment, which suggested that *S. aureus* might intend to reduce energy consumption by inhibiting protein biosynthesis. A similar process was also found in other exogenous agents, such as betulinaldehyde (Chung et al., 2013). On the other hand, genes involved in the TCA cycle and fatty acid degradation pathway, which are the main ATP-producing pathways in the cell (Wang S. et al., 2021; Qi et al., 2022), were up-regulated. These findings suggested that *S. aureus* intended to promote the TCA cycle and fatty acid degradation pathway to overcome the ATP deficiency induced by citral.

Activated TCA pathway not only supplies energy to the cell, but also generates ROS such as superoxide (O_2_•) and hydrogen peroxide (H_2_O_2_) through the electron transport chain (Kohanski et al., 2007). Excessive ROS will subsequently lead to oxidative stress occurs and rapid oxidation of various biological macromolecules, which is extremely harmful to the bacteria (Finkel and Holbrook, 2000). Bacteria produce several peroxidases for anti-oxidation, the most common of which are superoxide dismutase and catalase (Pacello et al., 2008). Here, after citral administration, the expression of genes encoding antioxidant enzymes such as catalase (*katA*), superoxide dismutase (*sodA*), and alkyl hydroperoxide reductase (*ahpC*) was up-regulated, indicating that citral might induce ROS overproduction in *S. aureus*. We also found the ROS content increased with the increasing of citral concentration in the ROS content assay. In contrast, the addition of thiourea, a ROS scavenger (Shee et al., 2022), can partially recover the growth of *S. aureus*. All these results indicated that citral could induce ROS overproduction and accumulation in *S. aureus*. OuYang et al. also found a similar massive accumulation of ROS when citral treated *Penicillium digitatum* (Ouyang et al., 2018).

The most significant target of ROS is believed to be DNA (Ha and Edwards, 2021). Transcriptomic analysis suggested that many genes (such as *uvrC*, *SAOUHSC_01469*) involved in DNA damage repair were up-regulated after citral treatment. Especially, *S. aureus* endonuclease III (encoding by *SAOUHSC_01469*) is shown to protect the bacterium against H_2_O_2_ stress (Barajas-Ornelas et al., 2014). The up-regulated DNA repair related genes suggested that ROS accumulation induced by citral might cause oxidative damage to DNA.

As we discussed above, the decreased ATP content caused by the citral subsequently induced the activation of the TCA pathway and increased of ROS content. The ROS then lead to DNA damage to inhibit the growth of *S. aureus*. The decrease of ATP content might be an important target of citral against *S. aureus*, which might be attributed to the suppressed expression of *atpD* and *atpG* genes.

The transcriptional analysis found that genes *vraS*, *vraR*, *and vraX*, which are responding to cell wall stress stimulon (Cuaron et al., 2013), were significantly enriched. Besides that, the genes (e.g., *plsY*, *scdA*, *tarA*) related to the phospholipids, cell wall and teichoic acid biosynthesis were repressed, which are important components of the cell membrane and cell wall (Rajagopal and Walker, 2017). All these results implied that citral might cause cell membrane and cell wall damages in *S. aureus*. Zhang et al. (2021) observed a similar result by the transmission electronic microscope citral could destroy the structures of the cell wall and cell membrane, causing leakage of cell contents.

The *Stahpyloccous aureus* infection pathway was also significantly enriched (*p*-adj < 0.05) in the down-regulated DEGs after citral treatment. Several genes involved in the biosynthesis of hemolysin, *Staphylococcus aureus* protein A, thermonuclease which are the virulence factors secreted by *S. aureus* and can help *S. aureus* to adhere to surface/tissues, evade immune system, and cause harmful toxin effects to the host (Singh and Phukan, 2019; Tam and Torres, 2019), and other virulence genes were down regulated after citral treatment. These findings indicated that citral might reduce the pathogenicity of *S. aureus* by down-regulating the expression of virulence factor-related genes.

**Figure 7:**
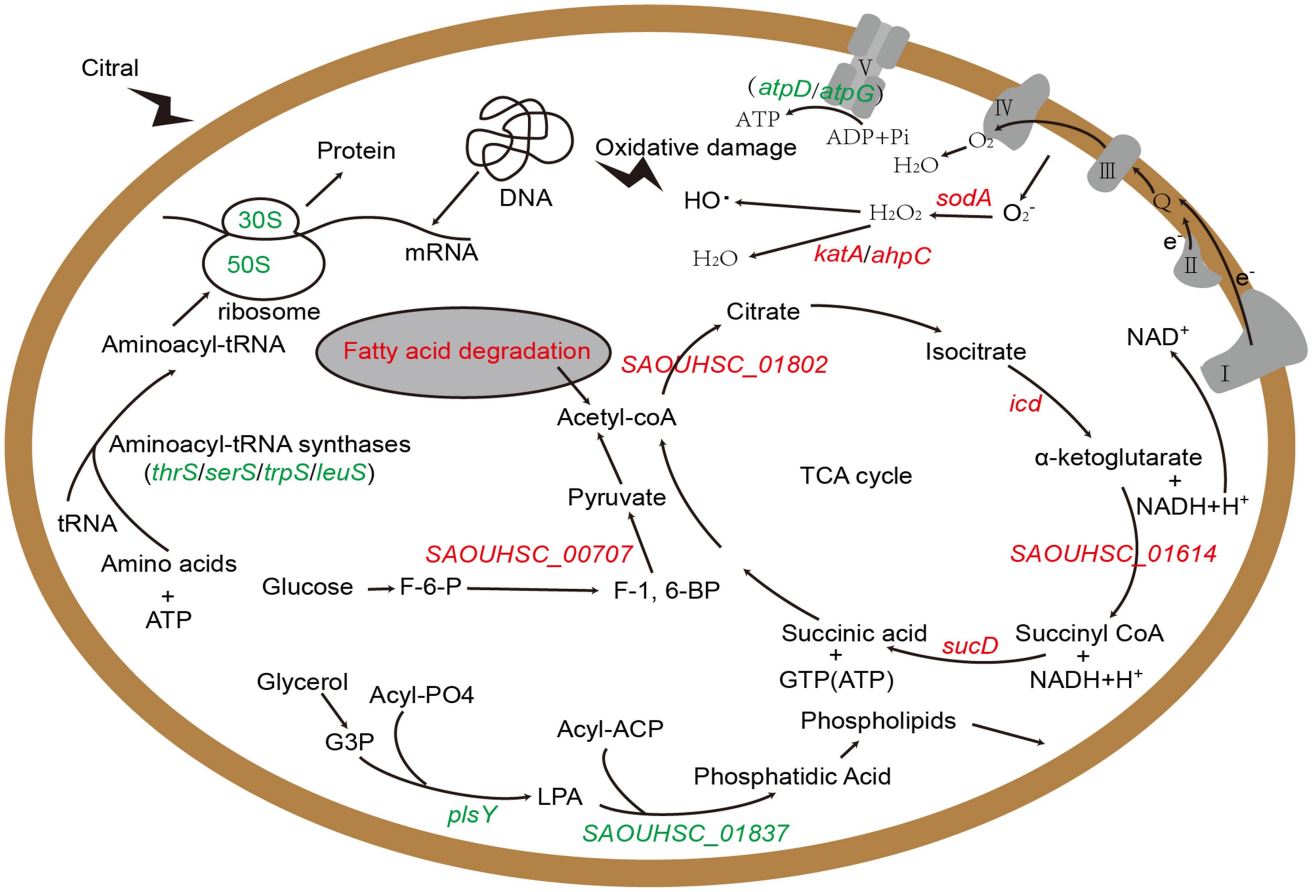
The schematic diagram of the mechanism of citral against *S. aureus* based on transcriptomic analysis. Red represents up-regulated genes and green represents down-regulated genes. G3P: Sn-glycerol-3-phosphate; LPA: 1-acyl-G3P; F-6-P: fructose-6-phosphate; F-1,6-BP: fructose-1, 6-bisphosphate.

Above all, we identified several key genes and metabolic pathways closely related to the antibacterial action of citral, which are vital for understanding the antibacterial mechanism of citral against *S. aureus*. The results of transcriptomic analysis and physiological trials indicated that citral might cause the reduction of ATP content by down-regulating ATP synthase genes (*atpD* and *atpG*), reduce the protein content, induce ROS accumulation and subsequent DNA damages, lead to cell membrane and cell wall damages, and reduce pathogenicity by down regulating virulence factor gene. As shown in Figure 7, the antibacterial mechanism of citral might be a multi-target mode that affects many molecular pathways in *S. aureus*.

## Data availability statement

The datasets presented in this study can be found in online repositories. The names of the repository/repositories and accession number(s) can be found at: https://ngdc.cncb.ac.cn/gsa, GSA: CRA009337.

## Author contributions

FS conceived and designed the project. ZL, KL, and WL performed experiment. GY and YS analyzed data. ZL and YX drafted the manuscript. FS and MD modified the manuscript. All authors contributed to the article and approved the submitted version.

## Funding

The study was supported by the grants from the Fund of the National Natural Science Foundation of China (Nos. 82102442, 32270449, and 31970137), the Sichuan Science and Technology Program (Nos. 2020JDRC0071 and 2021YJ0158), the Chengdu Medical College Graduate Student Innovation Fund (Nos. YCX2022-03-10, YCX2022-03-19), and Special Project of Traditional Chinese Medicine Research of Sichuan Provincial Administration of Traditional Chinese Medicine (No.2023ZD02).

## Conflict of interest

The authors declare that the research was conducted in the absence of any commercial or financial relationships that could be construed as a potential conflict of interest.

## Publisher’s note

All claims expressed in this article are solely those of the authors and do not necessarily represent those of their affiliated organizations, or those of the publisher, the editors and the reviewers. Any product that may be evaluated in this article, or claim that may be made by its manufacturer, is not guaranteed or endorsed by the publisher.

## Supplementary material

The Supplementary material for this article can be found online at: https://www.frontiersin.org/articles/10.3389/fmicb.2023.1171339/full#supplementary-material
