# Supplementary material for: Transcriptomic analyses reveal the potential antibacterial mechanism of citral against Staphylococcus aureus
Source: Front Microbiol. 2023 May 12;14:1171339. doi: 10.3389/fmicb.2023.1171339 (PMC10213633; doi:10.3389/fmicb.2023.1171339)
Supplement: Supplementary file 1 [file Data_Sheet_1.PDF]

Supplementary Material

Transcriptomic analyses reveal the potential antibacterial mechanism of citral against *Staphylococcus aureus*

Zedong Liao[1,2, †], Keshan Lin[1,2, †], Weijiang Liao[1], Ying Xie[1,2], Guoqing Yu[1,2], Yan Shao[3], Min Dai[1,2,*], and Fenghui Sun[1,2,*]

*Correspondence : sunfenghui@cmc.edu.cn (F.H.S); daimin1015@cmc.edu.cn (M.D)

1 Supplementary Figures

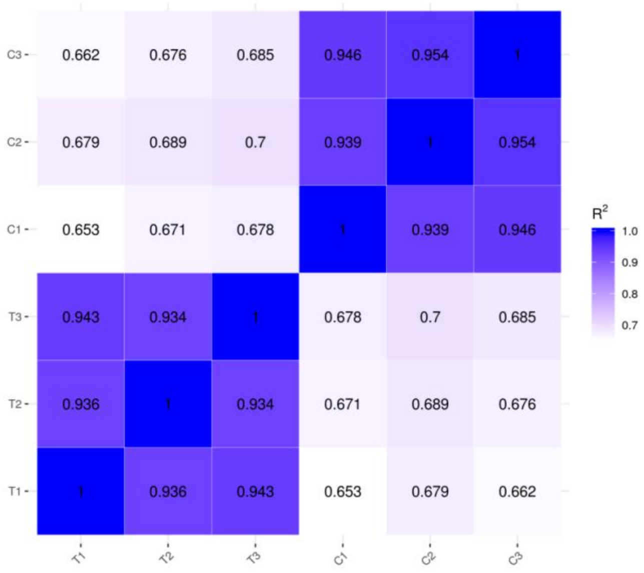

Supplementary Figure S1: Correlation analysis of RNA-Sequencing data. Correlation analysis of RNA-Sequencing data between citral-treated (T1-T3) and control (C1-C3) samples.

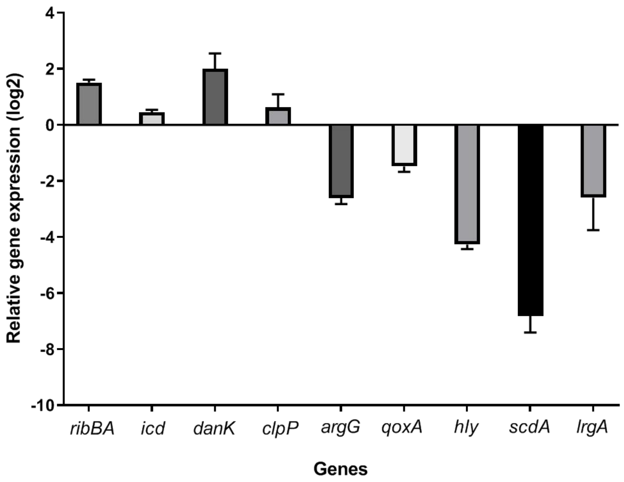

Supplementary Figure S2: The expression of the DEGs verified by qRT-PCR. The resulting data were derived from the average of three independent replicates.

2 Supplementary Tables

Supplementary Table S1. Primer sequences for qRT-PCR

Gene	Primer Sequence(5'-3')
tpi-F	CGTTGTTATCGGTCATTCT
tpi-R	TTACCACTTTCACGCTCTT
ribBA-F	AACTCCGACATCTTTGGTA
ribBA-R	ACGCCTTTATCTTGTGCTA
icd-F	TTTAGCTGCACAAGTTGGT
icd-R	TAATCTTATCTGCCGCTTC
dnaK-F	AAGCAACTAAAGATGCTGGTA
dnaK-R	CGAATACACCGTCACCTAA
clpP-F	CTGGTTTTGCGATTTATGA
clpP-R	TTCAGTTGCTTGTCCTTGA
argG-F	AGAGCGAATGAATGTGGTA
argG-R	CATCAATTTGAACTGGGAT
qoxA-F	TTGTTTCGTTGTACTTGGC
qoxA-R	TCTCACTCTTCGGTGGTTT
hly-F	AAAGTAGGCTGGAAAGTGA
hly-R	TAGCGAAGTCTGGTGAAAA
scdA-F	GTAACTGATTATCCGAAAGC
scdA-R	TTAACGAACCTGGTGTATT
lrgA-F	AAGACGCATCAAAACCAGC
lrgA-R	CTTCGCCTAACTTAACAGCAC

Supplementary Table S2. Basic statistical of RNA-sequencing data

Sample name	Raw reads	Clean reads	Raw bases	Clean bases	Q20(%)	Q30(%)	GC content(%)	Total mapping rate
T1	6806504	6768216	1.0G	1.0G	97.81	93.16	34.61	99.18%
T2	7527394	7492534	1.1G	1.1G	97.72	93.03	34.59	99.30%
T3	6674218	6638076	1.0G	1.0G	97.71	92.96	34.81	99.35%
C1	8172076	8118208	1.2G	1.2G	97.36	92.18	34.7	99.43%
C2	7740034	7696990	1.2G	1.2G	97.35	92.28	34.46	99.16%
C3	9005424	8949004	1.4G	1.3G	97.46	92.48	34.34	99.39%

Supplementary Table S3. The key DEGs in *Staphylococcus aureus* in response to citral.

gene_id	log2FoldChange	*p*adj	gene_name	gene_description
DEGs related to protein biosynthesis				
SAOUHSC_02505	-2.118404644	9.39E-31	rplP	50S ribosomal protein L16
SAOUHSC_02502	-2.29899961	2.20E-30	rplN	50S ribosomal protein L14
SAOUHSC_02500	-2.045158026	8.75E-29	rplE	50S ribosomal protein L5
SAOUHSC_02501	-2.101487346	1.82E-23	rplX	50S ribosomal protein L24
SAOUHSC_02509	-1.952550962	5.48E-23	rplB	50S ribosomal protein L2
SAOUHSC_02507	-2.144108413	1.91E-22	rplV	50S ribosomal protein L22
SAOUHSC_02495	-1.932482266	1.49E-20	rplR	50S ribosomal protein L18
SAOUHSC_02496	-1.672229283	1.69E-20	rplF	50S ribosomal protein L6
SAOUHSC_02512	-2.028617377	2.49E-20	rplC	50S ribosomal protein L3
SAOUHSC_02511	-1.918500333	4.54E-18	rplD	50S ribosomal protein L4
SAOUHSC_02510	-1.886638848	4.72E-16	rplW	50S ribosomal protein L23
SAOUHSC_02498	-2.086464235	8.80E-28	rpsH	30S ribosomal protein S8
SAOUHSC_02506	-2.153044204	2.26E-27	rpsC	30S ribosomal protein S3
SAOUHSC_02503	-2.07124287	3.50E-27	rpsQ	30S ribosomal protein S17
SAOUHSC_02508	-2.212730487	7.56E-27	rpsS	30S ribosomal protein S19
SAOUHSC_02499	-2.124789405	3.62E-26	rpsZ	30S ribosomal protein S14
SAOUHSC_02494	-1.833441637	1.66E-22	rpsE	30S ribosomal protein S5
SAOUHSC_02504	-2.127180083	6.92E-21	rpmC	50S ribosomal protein L29
SAOUHSC_02493	-1.566990154	1.33E-16	rpmD	50S ribosomal protein L30
SAOUHSC_01788	-1.683485435	1.75E-13	thrS	threonyl-tRNA synthetase
SAOUHSC_	-3.37594	1.29E-	serS	seryl-tRNA synthetase

00009	4991	46		
SAOUHSC_ 00933	-2.38782 4812	2.09E-23	trpS	tryptophanyl-tRNA synthetase
SAOUHSC_ 01875	-1.51027 644	7.26E-14	leuS	leucyl-tRNA synthetase
Glycolysis / luconeogenesis				
SAOUHSC_ 00707	1.83761 2794	4.7048 1E-21	-	1-phosphofructokinase
SAOUHSC_ 01430	2.67294 1414	1.84E-50	crr	PTS system transporter subunit IIA
SAOUHSC_ 00155	-1.50052 6448	1.55E-09	ptsG	PTS system glucose-specific protein
SAOUHSC_ 02848	-2.27424 0652	4.93E-33	glcB	PTS system glucose-specific transporter subunit IIABC
SAOUHSC_ 00206	-3.55712 5574	2.57E-43	ldh1	L-lactate dehydrogenase
SAOUHSC_ 00132	2.90664 6741	6.68E-43	aldA	aldehyde dehydrogenase
SAOUHSC_ 02822	1.56751 7831	4.66E-17	fbp	fructose-1,6-bisphosphatase
SAOUHSC_ 02614	-1.50269 0444	9.78E-12	-	aldose 1-epimerase
SAOUHSC_ 01794	-2.47003 8756	1.05E-29	gapA2	glyceraldehyde 3-phosphate dehydrogenase 2
SAOUHSC_ 00795	1.57082 2278	8.23E-21	gapA1	glyceraldehyde-3-phosphate dehydrogenase
SAOUHSC_ 01614	1.73962 1352	1.15E-25	-	dihydrolipoamide dehydrogenase
SAOUHSC_ 00608	-4.28754 727	1.06E-11	adh	alcohol dehydrogenase
SAOUHSC_ 00113	-1.66423 664	1.83E-10	-	bifunctional acetaldehyde-CoA/alcohol dehydrogenase
Citrate cycle (TCA cycle)				
SAOUHSC_ 01802	2.16986 849	4.10E-35	-	Citrate synthase
SAOUHSC_ 01347	2.83681 48	2.85E-44	acnA	aconitate hydratase
SAOUHSC_ 01801	3.34505 9953	3.60E-86	icd	isocitrate dehydrogenase
SAOUHSC_ 01614	1.73962 1352	1.15E-25	-	dihydrolipoamide dehydrogenase
SAOUHSC_ 01218	1.58533 9536	1.33E-18	sucD	succinyl-CoA synthetase subunit alpha
Fatty acid degradation				

SAOUHSC_00198	2.671676513	7.91E-53	-	AMP-binding enzyme
SAOUHSC_00196	2.227887249	3.20E-43	-	3-hydroxyacyl-CoA dehydrogenase
SAOUHSC_00132	2.906646741	6.68E-43	aldA	aldehyde dehydrogenase
SAOUHSC_00197	2.238791587	6.59E-37	-	Acyl-CoA dehydrogenase
SAOUHSC_00195	1.894535129	1.35E-31	-	acetyl-CoA acetyltransferase
Oxidative phosphorylation				
SAOUHSC_01065	-1.61089977	1.57E-16	-	heme A synthase
SAOUHSC_01066	-2.451828213	8.80E-34	ctaB	protoheme IX farnesyltransferase
SAOUHSC_02341	-1.688985611	1.02E-19	atpD	F0F1 ATP synthase subunit beta
SAOUHSC_02343	-1.737832017	5.00E-21	atpG	F0F1 ATP synthase subunit gamma
SAOUHSC_00999	-2.880460077	1.29E-50	qoxD	quinol oxidase subunit IV
SAOUHSC_01000	-2.957279653	2.29E-38	qoxC	cytochrome c oxidase subunit III
SAOUHSC_01001	-3.379276484	2.57E-70	-	quinol oxidase subunit I
SAOUHSC_01002	-3.974368281	3.59E-126	qoxA	quinol oxidase AA3 subunit II
DEGs related to ROS elevation and oxidative damage				
SAOUHSC_01327	2.724589655	3.17E-50	katA	catalase
SAOUHSC_01653	1.863522971	3.89E-20	sodA	superoxide dismutase dismutases, alpha-hairpin domain
SAOUHSC_00365	1.73333274	5.64E-14	ahpC	alkyl hydroperoxide reductase subunit C
SAOUHSC_00507	2.183455329	3.96658E-28	-	DNA repair protein
SAOUHSC_01469	2.220266289	2.35761E-36	-	endonuclease III
SAOUHSC_01102	1.629005042	2.45E-20	uvrC	excinuclease ABC subunit C
SAOUHSC_00564	-2.58794667	4.08E-06	ung	uracil-DNA glycosylase
SAOUHSC_01095	-1.793520741	0.004912975	rnhC	ribonuclease HIII

Homologous ecombination				
SAOUHSC_00445	1.856060198	3.29155E-21	recR	recombination protein
SAOUHSC_01179	1.558707754	9.95849E-20	-	primosomal protein N
SAOUHSC_01194	1.915446413	1.92086E-31	recG	ATP-dependent DNA helicase
DEGs related to cell membrance and cell wall				
SAOUHSC_01350	-2.642822666	6.71E-41	plsY	Glycerol-3-phosphate acyltransferase
SAOUHSC_01837	-3.08586999	5.88E-16	-	1-acyl-sn-glycerol-3-phosphate acyltransferase domain-containing protein
SAOUHSC_00229	-4.830603563	1.04E-103	scdA	cell wall biosynthesis protein
SAOUHSC_00640	-1.635133331	2.41E-08	tarA	teichoic acid biosynthesis protein
SAOUHSC_00561	9.040725294	0	vraX	C1q-binding complement inhibitor
SAOUHSC_02099	3.708805526	4.33E-71	vraS	histidine kinase
SAOUHSC_02098	3.724701453	1.64E-71	vraR	DNA-binding response regulator
DEGs related to virulence				
SAOUHSC_01121	-3.24706917	5.82E-38	hly	alpha-hemolysin
SAOUHSC_02260	-1.728991668	0.008969632	hld	delta-hemolysin
SAOUHSC_00069	-2.566380604	2.35E-24	spa	protein A
SAOUHSC_02963	-1.610958925	3.62E-13	clfB	clumping factor B
SAOUHSC_00544	-3.377981796	3.18E-60	sdrC	fibrinogen-binding protein
SAOUHSC_00545	-2.692515425	2.53E-34	sdrD	fibrinogen-binding protein
SAOUHSC_01114	-1.664054952	1.00E-05	fib	fibrinogen-binding protein
SAOUHSC_00818	-1.501883051	0.005981376	nuc	thermonuclease
SAOUHSC_01403	-2.498467652	7.89E-20	cspA	cold shock protein
SAOUHSC_02171	-2.421210189	3.66E-17	-	staphylokinase

SAOUHSC_00988	-4.076407744	2.63E-56	sspA	glutamyl endopeptidase
SAOUHSC_00987	-3.109238311	1.45E-54	sspB	cysteine protease
SAOUHSC_02706	-2.561092369	1.38E-21	sbi	immunoglobulin G-binding protein

Supplementary Table S4. The expression of the DEGs verified by qRT-PCR

gene_id	gene_name	log2FoldChange	$2^{-\triangle\triangle CT}$	trend
SAOUHSC_01887	ribBA	5.237528452	2.86±0.21	consistency
SAOUHSC_01801	icd	3.345059953	1.37±0.08	consistency
SAOUHSC_01683	dnaK	6.724355817	4.21±1.50	consistency
SAOUHSC_00790	clpP	4.043624152	1.60±0.51	consistency
SAOUHSC_00899	argG	-6.02947604	0.17±0.02	consistency
SAOUHSC_01002	qoxA	-3.974368281	0.36±0.05	consistency
SAOUHSC_01121	hly	-3.24706917	0.05±0.01	consistency
SAOUHSC_00229	scdA	-4.830603563	0.01±0.00	consistency
SAOUHSC_00232	lrgA	-3.055279106	0.20±0.16	consistency
