# Supplementary material for: Transcriptomic analyses reveal the potential antibacterial mechanism of citral against Staphylococcus aureus
Source: Front Microbiol. 2023 May 12;14:1171339. doi: 10.3389/fmicb.2023.1171339 (PMC10213633; doi:10.3389/fmicb.2023.1171339)
Supplement: Supplementary file 2 [file Table_1.DOCX]

| **Table 1.** The top ten up- and down- regulated DEGs. | | | | | |
| --- | --- | --- | --- | --- | --- |
| **Category** | **gene_id** | **log2Fold**  **Change** | ***p*-*adj*** | **gene_name** | **gene_description** |
| Up-  regulated | SAOUHSC_02893 | 9.707596956 | 6.929E-263 | - | DUF896 domain-containing protein |
|  | SAOUHSC_00561 | 9.040725294 | 0 | vraX | C1q-binding complement inhibitor |
|  | SAOUHSC_02892 | 8.903467143 | 0 | - | hypothetical protein |
|  | SAOUHSC_02866 | 7.489908404 | 0 | - | fatty acid efflux MMPL transporter |
|  | SAOUHSC_02872 | 7.334065647 | 4.3044E-201 | cwrA | cell wall inhibition responsive protein |
|  | SAOUHSC_01683 | 6.724355817 | 1.4228E-257 | dnaK | molecular chaperone |
|  | SAOUHSC_01684 | 6.604499429 | 4.8952E-205 | grpE | heat shock protein |
|  | SAOUHSC_02824 | 6.572427919 | 4.4638E-112 | - | alpha/beta hydrolase |
|  | SAOUHSC_00504 | 6.528347636 | 0 | mcsB | ATP:guanido phosphotransferase |
|  | SAOUHSC_00505 | 6.484127314 | 0 | clpC | endopeptidase |
| Down-  regulated | SAOUHSC_02671 | -6.471783081 | 1.611E-128 | narT | nitrate transporter |
|  | SAOUHSC_00899 | -6.02947604 | 3.46374E-83 | argG | argininosuccinate synthase |
|  | SAOUHSC_00188 | -5.983619288 | 2.0165E-102 | pflA | pyruvate formate-lyase 1 activating enzyme |
|  | SAOUHSC_00187 | -5.900147115 | 1.4499E-168 | pflB | formate acetyltransferase |
|  | SAOUHSC_02685 | -5.845924926 | 2.22966E-93 | - | sirohydrochlorin ferrochelatase |
|  | SAOUHSC_02681 | -5.770892267 | 8.0905E-128 | - | nitrate reductase subunit alpha |
|  | SAOUHSC_02680 | -5.655444057 | 1.3733E-110 | - | nitrate reductase subunit beta |
|  | SAOUHSC_00131 | -5.423736733 | 3.31075E-51 | - | YbaN family protein |
|  | SAOUHSC_02679 | -5.263068749 | 3.38926E-75 | - | respiratory nitrate reductase subunit delta |
|  | SAOUHSC_02645 | -5.222935859 | 2.92178E-05 | - | LytTR family DNA-binding domain-containing protein |
